# Efficacy of Ventriculoperitoneal Shunting Using Keen's Point Versus Frazier's Point in Patients With Hydrocephalus: A Non-Randomized Controlled Trial From Pakistan

**DOI:** 10.7759/cureus.81423

**Published:** 2025-03-29

**Authors:** Shehzad Safdar, Usama Mansoor, Asad O Omer, Tauqir Aslam Waraich, Ajlan Ali, Rabia Saleem

**Affiliations:** 1 Neurosurgery, Beaumont Hospital, Dublin, IRL; 2 Neurosurgery, Punjab Institute of Neurosciences, Lahore, PAK; 3 Urology, Lahore General Hospital, Lahore, PAK

**Keywords:** choudhary’s point, frazier's point, hydrocephalus, keen's point, optimal ventricular catheter tip position, pakistan, randomized controlled trial, ventriculoperitoneal shunt

## Abstract

Background

The ventriculoperitoneal (VP) shunt is crucial for draining excess cerebrospinal fluid, preventing complications like elevated intracranial pressure. Common points for the procedure, such as Keen's and Frazier's points, are established, with emerging interest in Choudhary’s point. Despite advancements, the challenge remains, as studies reveal cases with incorrectly placed catheter tips, emphasizing the ongoing need for improved ventriculostomy techniques.

Objective

To assess and compare the efficacy of VP shunting utilizing Keen's point versus Frazier's point in individuals diagnosed with hydrocephalus.

Methodology

From February to August 2022, this non-randomized controlled trial was conducted at Unit II, Department of Neurosurgery, Lahore General Hospital, Lahore. The study included 438 patients divided into two groups (Group A with Frazier's point and Group B with Keen's point), with 219 patients in each group, allocated based on the attending surgeon's preference. Effectiveness was evaluated post-procedure in the post-surgical ward using CT scans, focusing on factors such as optimal ventricular catheter tip position and minimal brain parenchymal damage. Data analysis utilized IBM Corp. Released 2012. IBM SPSS Statistics for Windows, Version 21.0. Armonk, NY: IBM Corp.

Results

In the Frazier's point group, the mean age was 18.47±9.21 years, while in the Keen's point group, it was 19.06±10.43 years. The duration of symptoms was 2.59±1.189 months in Frazier's point group and 2.65±1.237 months in Keen's point group. Gender distribution showed 42.5% males and 57.5% females in Frazier's point group and 40.6% males and 59.4% females in Keen's point group. The effectiveness of the VP shunting procedure was observed in 175 (79.9%) cases in Frazier's point group and 133 (60.7%) in Keen's point group (p <0.0001).

Conclusion

The study concludes that VP shunting at Frazier's point demonstrates superior effectiveness compared to Keen's point, supporting its consideration for hydrocephalus management across pediatric and adult populations.

## Introduction

The insertion of a ventriculoperitoneal (VP) shunt between the brain's ventricular cavity and the peritoneal cavity is crucial for draining excess cerebrospinal fluid, particularly in cases of ventricular obstruction or malabsorption [1-3]. Cerebrospinal fluid production in the ventricles at the choroid plexus underscores the need to prevent elevated intracranial pressure, which could lead to severe complications such as brain herniation and fatality [4-6].

The surgical procedure offers various points on the skull, notably Keen's point (3 cm above and 3 cm behind the pinna of the ear) [7-9] and Frazier's point (6 cm above the inion and 2.5 to 3.5 cm lateral to the midline) [10-12]. Choudhary’s point, situated 6 cm above and 3.5 to 4.5 cm lateral to the midline, gains attention for its increased effectiveness and lower complication rates [9,13,14]. Optimal ventricular catheter tip placement is considered crucial, emphasizing the need for improvement in the current techniques, as observed in 22.4 percent of cases where incorrect placements occur [15]. Previous studies comparing Keen's and Frazier's points report success rates of 78.6% and 87.5%, respectively, in VP shunt placements for hydrocephalus [8,16].

This study aims to systematically investigate and compare the efficacy of VP shunting using Keen's and Frazier's points, contributing valuable insights to optimize surgical techniques and provide evidence-based recommendations for neurosurgeons. The anticipated outcomes seek to enhance the understanding of optimal VP shunting, ultimately improving clinical outcomes and patient care.

## Materials and methods

Following approval from the Research Evaluation Unit of the College of Physicians and Surgeons Pakistan (CPSP) via the reference number CPSP/REU/NSG-2020-069-730 on February 18, 2022, this study adopted a non-randomized controlled trial design. The research was conducted at Unit II, Department of Neurosurgery, Lahore General Hospital, Lahore, spanning from February 2022 to August 2022. The determined sample size for the study was 438 cases. The calculation considered an 80% power of the study, a 5% significance level, and expected effectiveness rates of 87.5% [16] with Frazier’s point and 78.6% [8] with Keen’s point for VP shunt placement. The sampling technique employed was non-probability consecutive sampling.

Inclusion criteria

Patients within the age range of 14 to 80 years, both genders were diagnosed with hydrocephalus.

Exclusion criteria

Patients with evidence of infected cerebrospinal fluid (determined through CSF culture) and patients requiring shunt revision.

A total of 438 patients meeting the selection criteria were enrolled from the neurosurgery department's wards for this study. Informed consent was obtained, and demographic details, including age, gender, and symptom duration, were documented. Subsequently, the patients underwent the VP shunting procedure conducted by a single surgical team under general anesthesia with the researcher's assistance. The patients were allocated into two groups of 219 each based on the attending surgeon's discretion. Group A received a shunt placement using Frazier’s point, while Group B had shunts placed using Keen’s point. Following the procedure, patients were transferred to the post-surgical ward, where CT scans were obtained, and effectiveness was assessed in terms of the optimal position of the ventricular catheter tip within the brain ventricles, minimal damage to the brain parenchyma, and the absence of involvement of eloquent brain areas (e.g., the thalamus) in post-operative scans conducted 24 hours after the procedure. All relevant information was meticulously recorded on the attached proforma.

The gathered data underwent entry and analysis using IBM Corp. Released 2012. IBM SPSS Statistics for Windows, Version 21.0. Armonk, NY: IBM Corp. Quantitative variables, such as age and symptom duration, were expressed as mean and standard deviation, while qualitative variables like gender and effectiveness were presented as frequency and percentage. A chi-square test was utilized to assess the effectiveness between both groups, with a significance level set at p-value ≤ 0.05. The data was further stratified based on age, gender, and symptom duration. Following stratification, the chi-square test was employed to compare the effectiveness within each stratum, considering a significance threshold of p-value < 0.05.

## Results

The mean age in the Frazier's point group was 18.47±9.21 years, compared to 19.06±10.43 years in the Keen's point group. Regarding age distribution, 216 patients (98.6%) in Group A were within the 2-40 years range, and in Group B, 212 patients (96.8%) fell within the same range. Group A had three patients (1.4%) aged between 41 and 80 years, while in Group B, there were seven patients (3.2%) in the same age range. The duration of symptoms was 2.59±1.189 months in Frazier's point group and 2.65±1.237 months in Keen's point group. Gender distribution displayed 93 males (42.5%) in Group A and 89 (40.6%) in Group B, along with 126 females (57.5%) in Group A and 130 (59.4%) in Group B. Out of the total 438 patients, 175 (79.9%) in Group A (Frazier's point) and 133 (60.7%) in Group B (Keen's point) experienced effectiveness (p < 0.0001). The data underwent further stratification in both groups based on age, gender, and duration of symptoms. Within the 02-40 years age group, Frazier's point demonstrated significantly higher effectiveness (40.20%) compared to Keen's point (30.40%), with a p-value less than 0.0001. Conversely, in the 41-80 years age group, both Frazier's and Keen's points exhibited similar effectiveness at 30.00%, with a p-value of 0.091 (Table 1).

**Table 1:** Stratification for effectiveness in both groups with respect to age

Age group	Effectiveness	Frazier's Point Group	Keen's Point Group	Total	p-value
02-40 years	Yes	172	130	302	<0.0001
		40.20%	30.40%	70.60%	
	No	44	82	126	
		10.30%	19.20%	29.40%	
	Total	216	212	428	
		50.50%	49.50%	100.00%	
41-80 years	Yes	3	3	6	0.091
		30.00%	30.00%	60.00%	
	No	0	4	4	
		0.00%	40.00%	40.00%	
	Total	3	7	10	
		30.00%	70.00%	100.00%	

Regarding gender differences, a distinct pattern emerged, where Frazier's point displayed significantly higher effectiveness for males (42.90%) compared to Keen's point (29.70%) (p-value < 0.0001). Similarly, among females, Frazier's point exhibited notably higher effectiveness (37.90%) than Keen's point (30.90%) (p-value = 0.005) (Table 2).

**Table 2:** Stratification for effectiveness in both groups with respect to gender

Gender	Effectiveness	Frazier's Point Group	Keen's Point Group	Total	p-value
Male	Yes	78	54	132	<0.0001
		42.90%	29.70%	72.50%	
	No	15	35	50	
		8.20%	19.20%	27.50%	
	Total	93	89	182	
		51.10%	48.90%	100.00%	
Female	Yes	97	79	176	0.005
		37.90%	30.90%	68.80%	
	No	29	51	80	
		11.30%	19.90%	31.20%	
	Total	126	130	256	
		49.20%	50.80%	100.00%	

Analyzing the duration of symptoms, cases with a duration between 1 and 5 months showed significantly higher effectiveness at Frazier's point (40.00%) than at Keen's point (30.50%) (p-value < 0.0001). Conversely, for cases with a duration exceeding five months, there was no significant difference in effectiveness between Frazier's point (37.50%) and Keen's point (25.00%) (p-value = 0.465) (Table 3).

**Table 3:** Stratification for effectiveness in both groups with respect to the duration of symptoms

Duration of symptoms	Effectiveness	Frazier's Point Group	Keen's Point Group	Total	p-value
1-5 months	Yes	172	131	303	<0.0001
		40.00%	30.50%	70.50%	
	No	43	84	127	
		10.00%	19.50%	29.50%	
	Total	215	215	430	
		50.00%	50.00%	100.00%	
> 5 months	Yes	3	2	5	0.465
		37.50%	25.00%	62.50%	
	No	1	2	3	
		12.50%	25.00%	37.50%	
	Total	4	4	8	
		50.00%	50.00%	100.00%	

## Discussion

The current study compares the efficacy of VP shunting using Keen's point versus Frazier's point in hydrocephalus patients. The mean age in the Frazier's point group was 18.47±9.21 years, slightly lower than the Keen's point group, which had a mean age of 19.06±10.43 years. Age distribution revealed a higher percentage of patients in the 02-40 years range, with 98.6% in Group A and 96.8% in Group B. In the 41-80 years range, there were 1.4% in Group A and 3.2% in Group B (p=0.201). The distribution of duration of symptoms showed similar values for Frazier's point (2.59±1.189 months) and Keen's point (2.65±1.237 months) groups, with no significant difference (p=0.637). Gender distribution indicated 42.5% males in Group A and 40.6% in Group B, while females constituted 57.5% in Group A and 59.4% in Group B (p=0.698). In terms of effectiveness, Frazier's point exhibited a higher rate, with 79.9% effectiveness in Group A compared to 60.7% in Group B using Keen's point (p <0.0001). Overall, the study suggests a higher effectiveness with Frazier's point in hydrocephalus patients.

In a retrospective study conducted by Junaid et al. at Combined Military Hospital (CMH) Peshawar, ventriculoperitoneal shunting was carried out at Keen's point. The findings revealed positive clinical improvement in 114 patients, satisfactory outcomes in 20 patients, seven patient fatalities, and no observed change in two patients. The study concludes that the experience with VP shunting at Keen's point resulted in excellent outcomes, supporting its suitability for managing hydrocephalus [8].

In Woo et al.'s study involving 110 patients, the accuracy of ventricular cannulation at standard craniometric entry sites was compared. Keen's point demonstrated the highest correct identification rate (65%), followed by Kocher's point (65%) and Frazier's point (60%). The study concluded that Keen's point was the most accurate site, predicting optimal catheter position, and emphasized that catheter tip location, rather than the entry site, significantly influenced shunt survival [7].

Meybodi et al. proposed a novel burr hole point for posterior ventricular catheterization, positioned 51-57 millimeters posterior and 58-60 millimeters above the external auditory meatus parallel to the orbitomeatal plane. In comparison to classical Frazier and Keen points, this suggested point showed a significantly higher success rate (100% versus 83%, p = 0.052). Additionally, utilizing the suggested point may reduce the parenchymal mantle thickness, potentially enhancing the success of posterior ventricular catheterization [14].

Duong et al. conducted a retrospective review comparing ventriculoperitoneal shunt (VPS) approaches in adults for hydrocephalus management. The study included 93 patients, with 57% utilizing the occipital parietal point (OPP) and 43% using conventional landmarks. OPP showed lower rates of suboptimal placement (p = 0.0469) and a reduced likelihood of mechanical malfunctions (5.7% vs. 12.5%). The study suggests that OPP, identified on computed tomography (CT), is a safe alternative for optimal catheter positioning, potentially decreasing the risk of shunt malfunction due to suboptimal catheter placement [16].

The cumulative findings from these diverse investigations significantly enrich our understanding of ventriculoperitoneal (VP) shunting techniques, offering valuable insights into optimal approaches for hydrocephalus management. Notably, Frazier's point consistently emerges as a preferred and effective choice based on the evidence provided by our study and the corroborating studies by Junaid et al., Woo et al., Meybodi et al., and Duong et al. The retrospective analysis by Junaid et al. reinforces the commendable outcomes associated with VP shunting at Keen's point, aligning with our observations. The precision demonstrated by Keen's point in ventricular cannulation, as highlighted by Woo et al., underscores the importance of established craniometric entry sites. Meybodi et al.'s proposal of an alternative burr hole point for posterior ventricular catheterization, exhibiting promising success rates and potential advantages, adds a nuanced perspective to the discussion. Furthermore, Duong et al.'s comparative analysis, favoring the occipital parietal point (OPP) identified on computed tomography (CT), emphasizes the critical role of meticulous catheter positioning. In summary, the consistent preference for Frazier's point across these studies underscores its efficacy and positions it as a reliable choice in the ongoing refinement of VP shunting techniques for hydrocephalus management.

Limitations

Several limitations should be considered in this study. Firstly, the single-center setting may limit the generalizability of findings to diverse healthcare settings. The restricted 24-hour follow-up duration poses a challenge in assessing the sustained effectiveness and potential complications of VP shunting at Frazier's and Keen's points. The broad age range of 2 to 80 years may introduce inherent differences in patient characteristics. Addressing these limitations in future research is essential for a more comprehensive understanding of VP shunting efficacy.

## Conclusions

Considering the randomized nature of the study, we saw a superior effectiveness in VP shunting at Frazier's point compared to Keen's point. This suggests that Frazier's point may be considered for the management of hydrocephalus in both pediatric and adult populations. Frazier's point was also the preferred site of VP shunting for patients with shorter durations of symptoms. Disease-specific studies comparing both points are encouraged.
